# Developments in Screening Tests and Strategies for Colorectal Cancer

**DOI:** 10.1155/2015/326728

**Published:** 2015-10-04

**Authors:** Justin L. Sovich, Zachary Sartor, Subhasis Misra

**Affiliations:** ^1^School of Medicine, Texas Tech University Health Sciences Center, 1400 S. Coulter Street, Amarillo, TX 79106, USA; ^2^Department of Surgery, School of Medicine, Texas Tech University Health Sciences Center, 1400 S. Coulter Street, Amarillo, TX 79106, USA

## Abstract

*Background.* Worldwide, colorectal cancer (CRC) is the third most common cancer in men and second most common in women. It is the fourth most common cause of cancer mortality. In the United States, CRC is the third most common cause of cancer and second most common cause of cancer mortality. Incidence and mortality rates have steadily fallen, primarily due to widespread screening. *Methods.* We conducted keyword searches on PubMed in four categories of CRC screening: stool, endoscopic, radiologic, and serum, as well as news searches in Medscape and Google News. *Results.* Colonoscopy is the gold standard for CRC screening and the most common method in the United States. Technological improvements continue to be made, including the promising “third-eye retroscope.” Fecal occult blood remains widely used, particularly outside the United States. The first at-home screen, a fecal DNA screen, has also recently been approved. Radiological methods are effective but seldom used due to cost and other factors. Serum tests are largely experimental, although at least one is moving closer to market. *Conclusions.* Colonoscopy is likely to remain the most popular screening modality for the immediate future, although its shortcomings will continue to spur innovation in a variety of modalities.

## 1. Background

Worldwide, colorectal cancer (CRC) is the third most common cancer in men and second most common one in women [1]. It is also the fourth most common cause of cancer mortality [1]. Colorectal cancer (CRC) is the third most common cancer in the United States, as well as the second most common cause of cancer mortality [2, 3]. The lifetime prevalence of colorectal cancer in American men is 5% and only slightly lower in women [2]. Although those rankings have held steady in recent decades, incidence and mortality rates have steadily fallen [4].

The fall in CRC rates has not been even. Individuals over forty-nine years of age account for nearly the entire drop [5]. In fact, rates are rising in younger cohorts, although this population still accounts for a relatively low proportion of overall incidence (less than eight percent) [5]. Screening improvements, in methods and utilization rate, account for most of the drop in colorectal cancer burden [2]. Other important factors include changes in risk factors and improvements in treatment [2, 6].

Unfortunately, the primary risk factors for CRC remain poorly understood. The most compelling evidence for the role of lifestyle in CRC pathology is the stark difference in prevalence between developed and developing nations. Rates vary by factors of two and three, with higher disease burdens consistently limited to developed countries [1]. Rates in the United States are somewhat lower than in Europe and Australia but remain considerably higher than in other, less developed regions [1]. Moreover, immigrant populations experience a dramatic rise in colorectal cancer risk within one generation of moving to a developed economy [7].

Diet, fiber intake, and alcohol are all linked to colorectal cancer risk [8]. But the effect of each of these risk factors, alone and in the aggregate, is relatively subdued. They cannot account for disparities in colorectal cancer burden across countries [8]. Nor is the link between increasing CRC incidence in younger people and increasing obesity clearly established [2].

With the environmental basis for CRC still cloudy, screening remains the basis for colorectal cancer prevention. As with most cancers, early detection vastly improves prognosis. Five-year survival is nearly 90% for localized lesions and 70% for regional ones, but plummets to 13% with distant metastasis [4]. Depending on modality, the mortality benefit for CRC screening is somewhere between 25 and 50 percent of deaths prevented [9, 10]. Guaiac, endoscopic, and radiological methods are all available. Serum-based methods are advancing but remain investigative.

In the United States, there are at least three sets of screening guidelines (see Table 1). All three incorporate fecal occult blood, flexible sigmoidoscopy, or colonoscopy, alone or in combination. Colonoscopy is much more common in the United States than in other countries, even in other advanced economies. About two-thirds of Americans, 50 years and older, are compliant with USPSTF guidelines. A substantial majority meets those recommendations by colonoscopy [11, 12]. Lifetime colonoscopy prevalence in the United States is near 60% [13]. Data are sparser in Europe; lower GI endoscopy is only available as a primary screening tool in a handful of countries [13]. In Germany, about 3% of the eligible population appears to receive colonoscopy each year [9, 14]. Through 2008, 17.2% of eligible women and 15.5% of eligible men 55–74 years old had been screened [15].

Screening effectiveness depends on modality. Colonoscopy is the gold standard but still misses as many as 30% of adenomas [16]. Sigmoidoscopy does not visualize the proximal colon, from which forty percent of colorectal cancer arises [2]. Stool-based tests have low specificity and typically require follow-up sigmoidoscopy or colonoscopy.

Screening effectiveness also depends on rates of uptake and compliance. Intention-to-treat analysis and per-protocol effects often yield significantly different results [9]. Significant barriers discourage uptake of most of the major screening modalities, accounting in part for lower screening rates than in breast and cervical cancer. Embarrassment, lack of education, socioeconomic issues, concerns with masculinity, and cleanliness all play a role [3, 17]. Even the stool-based tests, considered less onerous by practitioners, seem unsanitary to the eyes of patients [17].

## 2. Stool-Based Screening

### 2.1. Guaiac-Based Fecal Occult-Blood Test (gFOBT)

Guaiac-based fecal occult-blood testing (gFOBT) was the first stool-based laboratory test used to screen for CRC. Two stool samples are placed on a test card, and hydrogen peroxide is applied. Guaiac, a plant resin, turns blue in the presence of hydrogen peroxide and a catalyst, heme. The test is purely qualitative and therefore operator-dependent [18].

gFOBT is the only noninvasive screening method that has prospective, interventional evidence demonstrating decreased CRC mortality. There have been five such trials, four in Europe and one in the United States. In the European trials, screening with gFOBT led to a 24–39% reduction in mortality in the study population and a 16–18% reduction in the general population [19–22]. The Mandel study, from the United States, demonstrated a 33% decrease in mortality for the study population [23]. The results from these trials are presented in Table 2.

gFOBT is inexpensive, requires few resources, and is ideal for large-scale community intervention. However, it has the lowest sensitivity of the noninvasive screens, ranging from 33% to 50% for CRC [24, 25]. Rehydration of samples prior to analysis, for instance, by the Hemoccult II Sensa (Beckman Coulter, Inc., Fullerton, CA), increases sensitivity for colorectal neoplasm, [26] but not to the levels of newer noninvasive testing. gFOBT is less likely to detect small adenomas, flat or sessile adenomas, and proximal lesions than larger, pedunculated, or distal ones [27]. It does, however, detect a greater proportion of villous and tubulovillous structures [27]. Certain meats, fruits, and vegetables may give false positive results. And because heme remains intact through the gastrointestinal (GI) tract, the test cannot distinguish between upper and lower GI bleed.

These issues have contributed to the declining popularity of gFOBT as a screening tool. It remains in the USPSTF guidelines, though, either as a yearly, primary modality or every three years in combination with sigmoidoscopy every five.

### 2.2. Fecal Immunochemical Testing (FIT)

Fecal immunochemical testing (FIT) also detects the presence of occult blood in stool but does so by agglutination of globin. Unlike heme, globin is degraded on its transit through the upper GI tract. This gives FIT increased specificity for lower GI bleed, without interference from dietary heme sources [28]. FIT is also more sensitive than gFOBT across all stages of CRC, from adenoma to advanced neoplasia [29–35]. Detection rates are 1.5–2.5 times higher than gFOBT for CRC and 2–4 times higher for advanced neoplasia.

A major advantage of FIT is its quantitative interpretation of globin levels. Unlike gFOBT, which is purely qualitative, FIT analysis can be automated and the results standardized, reducing operator error. Positive test result cutoff points have been well studied. Sensitivities at the lowest cutoff values, 20–50 ng/mL, range from 66% to 88% for CRC detection [34–36], while sensitivity at the highest cutoff value of 300 ng/mL is 56% for CRC [37]. The most common cutoff value studied is 100 ng/mL with sensitivities ranging from 60% to 82% for CRC [25, 38, 39]. As with most lab tests, specificity, and sensitivity correlate inversely, and the ideal cutoff number depends on population characteristics and the availability of confirmatory testing.

The performance of FIT as a function of polyp morphology appears broadly similar to gFOBT, [27, 40] despite an early study appearing to show similar performance across different polyp types [41].

The available evidence supports the superiority of FIT over gFOBT, but there are no interventional studies yet to demonstrate mortality reduction. Based on its predicted benefit, the American College of Gastroenterology recommends it over gFOBT for nonendoscopic screening of CRC.

### 2.3. Fecal DNA Testing (Cologuard©)

Multitarget fecal DNA testing has been an area of increased research interest over the last several years. The FDA recently approved the first commercially available test, marketed as Cologuard© (Exact Sciences, Madison, WI). Exfoliated CRC cells turn over more quickly than healthy cells and are shed in the stool. Cologuard© targets gene mutations associated with these cancerous cells. These include aberrantly methylated BMP3 and NDRG4, seven different point mutations in KRAS, *β*-actin gene (a reference for human cells), and hemoglobin [42].

Imperiale et al. published the leading study on Cologuard© in the New England Journal of Medicine in 2014 [43]. The study randomized subjects to Cologuard© or FIT screening. Cologuard© sensitivities were superior to those of FIT for every type of pathologic lesion, albeit at somewhat lower specificity (Table 3).

Other studies have extended Imperiale's findings to additional gene mutations, with sensitivities ranging from 85% to 100% for detection of CRC and 53% to 83% for high-grade dysplasias and adenomas [44–46]. And, in a subsequent paper, Imperiale et al. have demonstrated Cologuard© superiority to gFOBT [47].

Cologuard's lower specificity relative to FIT and gFOBT could result in greater numbers of follow-on colonoscopy. The cost-benefit consequences are yet to be determined.

## 3. Endoscopic Screening

The three major endoscopic screening methods (see Table 4) differ chiefly in the extent of bowel visualized. So, for instance, the 45% sensitivity of flexible sigmoidoscopy for polyp detection reflects the fact that it cannot visualize the proximal colon, where nearly half of colorectal cancer arises.

### 3.1. Rigid and Flexible Sigmoidoscopy

Sigmoidoscopy is the oldest and most thoroughly researched of the endoscopic methods. Rigid sigmoidoscopy has existed for a century and flexible sigmoidoscopy for about half as long [48]. Its chief advantages are its simplicity and wide availability [49]. It can be performed by nonspecialists and, in some cases, nonphysicians, which makes it especially useful in the developing world [49]. Relative to colonoscopy, it requires less preparation, an enema, usually, instead of a thorough bowel cleanse [49, 50]. However, sigmoidoscopy is uncomfortable for the patient and, as its name implies, does not visualize the proximal colon.

Sigmoidoscopy appears to decrease colorectal cancer incidence by somewhere between twenty and thirty percent, by intent-to-treat analysis [49, 51]. This number increases to between forty and fifty percent for patients actually treated, demonstrating the prominent role of compliance in this type of screening [49]. In another quirk of compliance factors, patients screened by sigmoidoscopy have significantly poorer health habits after the screen than their unscreened counterparts [52].

### 3.2. Colonoscopy

Colonoscopy is both the gold standard for colorectal cancer screening and by far the most common means of colorectal cancer screening in the United States. Colonoscopy reduces the odds of colorectal cancer by somewhere between thirty and seventy-five percent [50]. Risk reduction remains unknown; the only major, randomized controlled studies are still in progress [50].

Colonoscopy is not without shortcomings. It misses about twenty to thirty percent of adenomas [16, 51]. The procedure is relatively complex, which introduces uncertainty. The quality of the colonoscopy depends on bowel preparation and operator characteristics [51]. Smaller, flatter lesions are more difficult to visualize and are missed more often than larger, pedunculated ones [53]. In a prospective study by Heresbach et al., 29% and 32% of sessile and flat polyps, respectively, were missed, compared with just 5% of pedunculated lesions [53]. Unfortunately, flat lesions are also particularly likely to be cancerous [54, 55]. Some studies have suggested that even though colonoscopy can visualize the proximal colon, it is less adept at finding lesions there than in the distal portion [55]. The complexity of colonoscopy leads to wide differences in operator proficiency. Adenoma detection rate, the best validated quality metric, ranges from 7% to 44% depending on the endoscopist [56].

The best-validated metric for colonoscopy effectiveness is the adenoma detection rate (ADR), the proportion of screened subjects in whom at least one adenomatous lesion is identified [16, 56]. Higher ADR correlates with fewer interval cancers [51]. Less is known about how ADR is related to incidence and mortality. However, most postcolonoscopy colorectal cancers are thought to be due to missed lesions, rather than new ones, which makes ADR a valuable measurement [16]. Current guidelines suggest that the ADR should be at least 25% in men and 15% in women [55]. Other, unverified metrics include endoscope withdrawal time intubation rate, bowel preparation, patient comfort, sedation, and complication rates [16].

A long list of colonoscopy enhancements have been attempted, some with more promise than others. High-definition white-light colonoscopy does not appear to offer much benefit over standard methods [51, 55, 57]. Chromoendoscopy is another advanced visualization technique that uses a dye spray to enhance contrast. It has shown more promise than high-definition imaging, but only in high-risk groups like IBD patients [51, 55, 58].

Another visualization method with doubtful benefit is cap-assisted colonoscopy. This technique uses a transparent cap at the tip of the colonoscope to improve visualization and depress mucosal folds. The balance of studies suggests no benefit to cap-assisted colonoscopy, or perhaps a small benefit confounded with the greater withdrawal times associated with this method [51, 55, 59, 60].

A more promising technical solution is the “third-eye retroscope,” a colonoscope with an elaborated camera giving simultaneous anterograde and retrograde views of the colon [51, 55, 61, 62]. Multiple studies have demonstrated improvements in polyp detection rates. At least one of these studies, though, also showed the TER withdrawal time was two minutes longer than control, a metric independently associated with higher adenoma detection rate. And no gadget comes free: a third-eye retroscope processor costs about $20,000 and the disposable catheter another $350 [51, 63].

While technology proceeds in fits and starts, several low-tech measures have proven effective at improving colonoscopy performance. A clean colon is an easy to inspect colon, so proper bowel preparation is essential [55]. The process is unpleasant; innovations that make it less unpleasant have value. Low-volume preparations are more effective than high-volume preparations, and it helps to split the preparation into two doses [55]. Finally, nurse assistance with spotting polyps increases the number of polyps found [55].

### 3.3. Capsule Endoscopy

In capsule endoscopy, the patient swallows a pill-sized camera that wirelessly transmits images as it tumbles through the gut. The patient's bowel motility determines the capsule's progress, and the capsule's arbitrary orientation dictates the image captured by the camera. This lack of operator control currently limits capsule endoscopy to small bowel inspection, due to that segment's relative inaccessibility to other endoscopic methods [64]. Research is ongoing, though, for its using as a CRC screening tool, particularly as the technology improves. The most recent research is limited to the second generation of the capsule, while the third generation has only been available since 2013. Successive generations have improved frame rates and resolution.

At least three studies have measured sensitivities and specificities for capsule endoscopy, typically only for medium- to larger-sized polyps, from ≥6 mm to ≥10 mm. These studies have found, for 6 mm polyps, sensitivities between 84% and 89% at 64% to 82% specificity [64–66]. Both numbers expectedly improve for larger polyps: 88% sensitivity at specificities ranging from 89% to 95% for polyps greater than 10 mm in size.

## 4. Radiology-Based Screening

### 4.1. Contrast Barium Enema

Contrast barium enema was the first radiological technique for examining the colon for structural lesions. There are two primary methods: single contrast and double contrast (SCBE and DCBE, resp.). SCBE uses barium alone, while DCBE relies on air contrast in addition to barium. An experienced radiologist must actively manipulate the colon during fluoroscopy to evenly distribute barium and provide imaging adequate for interpretation.

SCBE is 59% sensitive for polyps in a CRC screening population [67]. Barium contrast alone tends to obscure certain lesions, making study results more difficult to interpret. DCBE on the other hand is 87% sensitive for all polyps [67] and 96% sensitive for polyps >1 cm [68]. DCBE's screening value is comparable to that of colonoscopy [69]. In fact, DCBE may detect more of the very proximal colon lesions that colonoscopy sometimes misses [70].

SCBE and DCBE have largely fallen out of favor despite the evidence supporting their utility, and many radiologists no longer perform this study. Newer imaging options, such as computed tomography (CT) and magnetic resonance imaging (MRI), offer similar or superior performance without operator dependence. Because of its effectiveness as a screening tool, Medicare still reimburses barium contrast fluoroscopy, and it still has a role for patients in whom other radiologic methods might be contraindicated.

### 4.2. CT Colonography

Computed tomography (CT) for CRC screening, known as CT colonography or virtual colonoscopy, is a newer use for a popular imaging tool. Computer software constructs 2D and 3D images of the colon from CT-scan data. Sensitivity for polyps >9 mm is between 85% and 93% with a specificity of 97%, according to two meta-analyses (see Table 5) [71–73]. This decreases to 70% for polyps 6–9 mm and 48% for polyps <6 mm [73]. An additional study by Macari et al. shows sensitivity to range from 12% for polyps of 5 mm or less but 70% and 93% for polyps 6–9 mm and greater than or equal to 10 mm, respectively [74]. Those figures are comparable to endoscopic colonoscopy and superior to DCBE [75].

Various radiological advances have improved the performance of CT colonography. Multidetector scanners are 7% more sensitive than single detectors [73]. They are also faster and less irradiating. Helical CT for colonography has been shown to be 100% sensitive for polyps of at least 10 mm, 83.3% sensitive for polyps 6–9 mm, and 51.3% sensitive for those 5 mm or less [76]. Tagging agents increase test performance by marking stool and fluid collections left over after preparatory colon cleanse. These collections can mimic polyps or obscure them, causing false negatives. Oral barium tags stool and residual solids, while oral iodine tags residual fluids. These oral agents have proven effective in clinical trials, the most notable being the ACRIN National CT Colonography Trial [72, 77].

With the routine use of multidetector scanners and oral tagging agents, CT colonography has become increasingly sensitive for polyps. However, radiologists and other healthcare providers must still interpret study results. Computer-aided diagnosis (CAD) is a useful interpretive adjunct in mammography and other radiological studies. Likewise, it increases test sensitivity for CT colonography when used in addition to radiologist interpretation, [78, 79] and is 90.1% sensitive for all polyps when used as the sole interpreter [80].

The performance of CT colonography relative to screening colonoscopy is not yet clearly established. A large-scale trial by Pickhardt et al. showed CT colonography to be superior to colonoscopy for polyps larger than 1 cm, with a sensitivity of 94% versus 88% [81]. However, colonoscopy was more sensitive for polyps 6 mm and larger, with a sensitivity of 92% versus 89% [81]. An additional study by Gluecker et al. demonstrates that multidetector CT colonography is 90% specific compared to gold standard colonoscopy [82]. Although the tests seem to have similar performance characteristics, one study by Pedersen et al. shows that CT colonography may be present more of a technical challenge since the study specific relies on absence on artifacts which are easily found in radiologic imaging [83]. The differences between the two tests appear to be small.

The relative sensitivity of CT colonography and other radiologic methods for flat and sessile polyps is also controversial. Intuitively, a screening modality that visualizes shapes should perform less well with lesions that have less shape. Lesion morphology is not considered in the Pickhardt trial mentioned above [81]. The Gluecker trial does not systematically consider flat lesions but does mention that a flat lesion was missed due to “perceptive error” [82]. Elsewhere, Pickhardt has found sensitivities of between 80% and 83% of CT colonography for flat lesions (for adenomas and flat lesions greater than 6 mm, resp.) [84]. Of course, these values were obtained against a reference standard of optical colonoscopy, which will itself miss a greater number of flat polyps than sessile or pedunculated ones. Other studies have found sensitivities as low as 50% [85]. Still, flat lesions are not usually entirely flat (they are typically defined as having height less than 1/2 or 1/3 of width), and they have a different attenuation than surrounding fat [82, 84]. Contrast coating the surface of these polyps increases the sensitivity of CT colonography for them [86]. But, including more suspected flat lesions also depresses the positive predictive value of CT colonography, from 96.5% and 92.5% for pedunculated and sessile polyps to 77.7% for flat [87].

Although CT colonography performance is comparable to colonoscopy, concerns over radiation exposure and cost have hindered its widespread adoption. The USPSTF cites radiation dosing for its decision not to recommend CT colonography. CT colonography remains, though, in the screening recommendations of the American College of Gastroenterology and the American Cancer Society. CT colonography is also more expensive than colonoscopy, and neither Medicare nor Medicaid covers the cost of the test. Without further improvements to these drawbacks or a clear demonstration of superior performance, CT colonography may remain merely promising or an alternative for appropriate patients.

### 4.3. MRI Colonography

MRI colonography works similarly to CT colonography. MRI data generates a virtual representation of the colon. Two techniques are used: light lumen and dark lumen. Light lumen MRI colonography uses liquid enema with gadolinium contrast, while the dark lumen method relies on air or gas contrast enema with IV contrast. Polyps appear as a filling defect on bright lumen MRI and as an enhancing lesion on dark lumen MRI.

MRI colonography is relatively new, and the few studies that examine its performance vary widely in their criteria and results. In one study, sensitivities are 38% for polyps 6–9 mm, [88] while others show 84% and 100% for polyps of similar size [89, 90]. A meta-analysis by Zijta et al. concludes that MRI colonography has 88% sensitivity at 99% specificity for polyps 10 mm and larger [91]. Studies comparing MRI colonography to colonoscopy have shown sensitivities ranging from 93% to 100% for polyps 6–9 mm. [92–94]

The relative performance of MRI colonography for different polyp shapes is unknown. The trials listed above do not discriminate between lesion shapes, although Luboldt speculates that flat lesions “will likely remain obscure” on MRI [93]. Subsequent studies appear limited anecdotal accounts of missed flat lesions [95–97].

Unlike its CT counterpart, MRI colonography does not use radiation. However, MRI is more expensive and is contraindicated for patients with prostheses, pacemakers, or other metal implants. As with CT colonography, data supporting its superiority to the gold standard colonoscopy are lacking. More data are needed, though, before a definitive judgment can be rendered.

## 5. Serum-Based Screening

Currently, “serum-based screening” barely exists. The research is extensive, but little of it is validated and even less commercialized [18]. Indeed, most of the present literature is limited to colorectal cancer detection, instead of adenoma detection. But as with capsule endoscopy, the field is young, and at least some of the innovations are promising. In general, researchers have approached the problem from two directions: first, to try to identify novel, more powerful biomarkers, and second, to try to combine known biomarkers by algorithm to find patterns suggestive of colorectal cancer.

CEA is the traditional marker associated with colorectal cancer. Alone, its sensitivity for colorectal cancer disease is about 40%, too low to use as a screen, but valuable as a tool to monitor cancer recurrence, where its sensitivity doubles to 80% [98]. At least two groups have combined CEA with other markers associated with colorectal cancer, like ferritin and seprase, using multivariate analysis to yield sensitivities between 65.8% and 68% for colorectal cancer detection [32, 99]. This is comparable to FIT, although not yet as cost effective [99]. The adenoma detection rates for these multivariate analyses remain too low to be useful, 22.7% in the Wild study [99].

The most studied alternative to CEA is methylated septin-9, an epigenetic modification associated with colorectal cancer [18]. Studies vary widely on its predictive value, with numbers ranging from 48% to 90% sensitivity for colorectal cancer [18, 100]. Again, these values are considerably lower for adenomas (≥1 cm), ranging from 11% to 29%. These data were sufficient for approval from the European Medicines Agency (the Epigenomics Epi proColon), but an FDA advisory panel split 5-4 on the matter in early 2014, and the FDA itself has requested further data on screening compliance before granting approval [101].

Other DNA, RNA, and protein molecules under study tend to track the same markers studied in stool. For instance, guanylyl cyclase RNA has a sensitivity of 74% at 95% specificity for CRC [99]. And p53 autoantibodies have low sensitivity (around 25% at 95% specificity for CRC) but might be used in combination with other markers [102]. The full breadth of these efforts is outside the scope of this review: Imperiale gives a good summary [18]. An interesting issue will be whether it is more effective to measure these markers from stool or from blood. The most recent study, from Ahlquist et al., detected a greater number of adenomas from stool DNA than from plasma [103]. The few earlier studies are split [104, 105]. Even if plasma-based assays are less powerful, they may benefit from greater patient compliance. While Cologuard bills itself as the first “at home” screen, there is at least some evidence that patients find sampling their own feces more objectionable than do physicians [17]. And, while patients may dislike needles, blood testing is a standard feature of medical practice.

Finally, it should be stressed that most of the research in serum-based markers has been for CRC detection, not adenoma detection. And, as noted above, the studies that have included adenoma in their data have showed a wide gap in the ability of serum-based markers to detect these early lesions.

## 6. Discussion and Conclusions

The European Commission set out its goals for cancer screening, including CRC screening, in a 2003 Council Recommendation, mentioning only fecal occult-blood testing [106]. Two subsequent reports on the implementation of these recommendations have identified shortcomings. The first, in 2008, found fewer than half the minimum number of screenings had taken place [107]. The second, in 2014, found that screening programs had only been implemented in 15 of 25 countries [108]. With the exception of Germany, at 54.2% of eligible people screened, no other country had more than a 26% screening rate for CRC [109]. Among the EU countries covered by the report, colonoscopy is available as a primary screening modality only in Germany, Austria, Poland, and the Czech Republic [13].

In the United States, the National Colorectal Cancer Screening Roundtable has a stated goal of an 80% colon-cancer screening rate by 2018. In contrast to most of Europe, the screening rate in the United States already approaches two-thirds of the eligible population. Germany approaches this rate, but colonoscopy remains much more frequently used in the United States. Current guidelines give a range of options for effective screening, which this paper summarizes.

To a greater extent than with other cancers, barriers to screening alter the effectiveness of the various colorectal cancer screening methods. The best test is often the one that patients will do. In the United States, the CRC screening rate, although relatively high, may lag the cervical and breast cancer screening rates because of the relatively high barriers to existing modalities. Another shortcoming shared by all of the available screening methods is sensitivity for small, flat, and sessile polyps. There has been particular concern on this point with radiologic screening, but these types of polyps bleed less and are more difficult to see even under direct visualization, and all available screening modalities perform substantially less well.

Stool-based tests are less sensitive than colonoscopy, but improving. New advances, like the Cologuard test, catch significantly more polyps than occult-blood tests. Although less popular than colonoscopy, stool-based tests continue to be used as the primary screen in a substantial minority of patients. Their technical shortcomings are at least in part overcome by their practical ones: their simplicity and lower cost mean that they might be used by a greater part of the population more frequently than is possible with colonoscopy.

Colonoscopy is the gold standard colorectal cancer screen, the first, best option, and most common screen in the United States. As with cervical and breast cancer screening, its widespread implementation has substantially decreased the CRC burden in this country. But its utilization still lags the Pap smear and mammogram, and its costs and complexity are greater than either of those two tests. Barriers to colonoscopy uptake and shortcomings in the test itself continue to encourage research on alternative modalities.

The only screens that consistently rival colonoscopy in power are the radiological ones, CT and MRI colonography, in particular. Their problems reverse those of the stool-based tests: for all their technical merit, they remain expensive and, in the case of CT colonography, carry a small, but real radiation risk. Positive screens require follow-up colonoscopy to remove the identified polyps. Until ease of use improves or they show significantly better performance than colonoscopy, radiological screens may remain a minor alternative to endoscopic and noninvasive screens.

## Figures and Tables

**Table 1:** Screening recommendations (adapted from Short et al. [110]).

Organization	Screen and interval	Age
United States Preventive Services Task Force (USPSTF)	(1) Fecal occult blood (1-year) (2) Flexible sigmoidoscopy (5-year) + high-sensitivity FOBT (3-year) (3) Colonoscopy (10-year)	50+ years

American College of Gastroenterology	*Preferred* (1) Colonoscopy (10-year) (2) FIT (1-year), if colonoscopy declined *Alternative (prevention)* (3) Flexible sigmoidoscopy (5- to 10-year) (4) CT colonography (5-year) *Alternative (cancer detection)* (5) gFOBT (1-year) (6) Stool DNA (3-year)	45+ years for blacks 50+ years everyone else

American Cancer Society	*Adenoma and cancer* (1) Flexible sigmoidoscopy (5-year) (2) Colonoscopy (10-year) (3) Double-contrast barium enema (5-year) (4) CT colonography (5-year) *Cancer* (5) High-sensitivity FOBT (1-year) (6) FIT (1-year) (7) Stool DNA (Cologuard) (uncertain)	50+ years

**Table 2:** Interventional clinical trial with gFOBT.

Study	Study population mortality reduction	General population mortality reduction
Kronborg et al. (1996) [19]	39%	18%
Hardcastle et al. (1996) [20]	33%	16%
Faivre et al. (2004) [22]	33%	15%
Lindholm et al. (2008) [21]	24%	16%
Mandel et al. (1993) [23]	33%	—

**Table 3:** Sensitivity and specificity of Cologuard compared to FIT (from Imperiale et al. [43]).

Sensitivity		
Lesion detected	DNA	FIT
CRC	92.3%	73.8%
Advanced precancerous^*∗*^	42.4%	23.8%

Specificity		
Lesion type and colonoscopy result	DNA	FIT

Nonadvanced w/negative scope	86.6%	94.9%
Negative scope	89.8%	96.4%

^*∗*^Advanced precancerous lesions include “advanced adenomas (high-grade dysplasia or with ≥25% villous histologic features or measuring ≥1 cm in the greatest dimension) and sessile serrated polyps measuring 1 cm or more in diameter.”

**Table 4:** Performance of endoscopic colonoscopy screens.

	Sensitivity	Advantages	Disadvantages	Comments
Rigid sigmoidoscopy	~25% [54]^*∗*^	Ease of operation Can be performed by clinician	Patient discomfort Does not visualize proximal colon	Rarely used in USA

Flexible sigmoidoscopy	~45% [54, 111]^∧^	More comfortable than rigid	Does not visualize proximal colon	Rarely used in USA

Colonoscopy	~80% [16, 111]^∧^	Gold standard	Extensive bowel preparation No randomized controlled prospective studies	

^*∗*^Reported as polyps detected.

^∧^Sensitivity for ≤5 mm polyps; the figures for CRC are 60 and 95%, respectively.

**Table 5:** Performance of CT colonography.

	Sensitivity	Specificity
Single detector	59–92%	82–98%
Multidetector	82–100%	90–98%
Pooled via meta-analyses	85–93%	97%
